# The Impact of Dextran Sodium Sulfate-Induced Gastrointestinal Injury on the Pharmacokinetic Parameters of Donepezil and Its Active Metabolite 6-*O*-desmethyldonepezil, and Gastric Myoelectric Activity in Experimental Pigs

**DOI:** 10.3390/molecules26082160

**Published:** 2021-04-09

**Authors:** Jan Bures, Ilja Tacheci, Jaroslav Kvetina, Vera Radochova, Lukas Prchal, Darina Kohoutova, Martin Valis, Martin Novak, Rafael Dolezal, Marcela Kopacova, Stanislav Rejchrt, Vit Sestak, Veronika Knoblochova, Eva Peterova, Jana Zdarova Karasova

**Affiliations:** 1 2nd Department of Internal Medicine-Gastroenterology, Charles University Faculty of Medicine in Hradec Kralove and University Hospital, 500 03 Hradec Kralove, Czech Republic; tacheci@gmail.com (I.T.); kvetina.jaroslav@seznam.cz (J.K.); darina.kohoutova@seznam.cz (D.K.); marcela.kopacova@fnhk.cz (M.K.); rejchrt@lfhk.cuni.cz (S.R.); veronika.knoblochova@fnhk.cz (V.K.); PETEROVE@lfhk.cuni.cz (E.P.); 2 Animal Laboratory, Faculty of Military Health Sciences, University of Defence, 500 01 Hradec Kralove, Czech Republic; vera.radochova@unob.cz; 3 Centre of Biomedical Research, University Hospital, 500 05 Hradec Kralove, Czech Republic; lukas.prchal@fnhk.cz (L.P.); martin.novak@fnhk.cz (M.N.); rafael.dolezal@fnhk.cz (R.D.); zdarova.jana@gmail.com (J.Z.K.); 4 The Royal Marsden Hospital NHS Foundation Trust, London SW3 6JJ, UK; 5 Department of Neurology, Charles University Faculty of Medicine in Hradec Kralove and University Hospital, 500 03 Hradec Kralove, Czech Republic; martin.valis@fnhk.cz; 6 Department of Pharmaceutical Chemistry and Pharmaceutical Analysis, Charles University Faculty of Pharmacy, 500 05 Hradec Kralove, Czech Republic; 7 Department of Clinical Biochemistry and Diagnostics, University Hospital Hradec Kralove, 500 05 Hradec Kralove, Czech Republic; vit.sestak@fnhk.cz; 8 Department of Toxicology and Military Pharmacy, Faculty of Military Health Sciences, University of Defence, 500 01 Hradec Kralove, Czech Republic

**Keywords:** 6-*O*-desmethyldonepezil, dextran sodium sulfate, donepezil, electrogastrography, experimental pigs, gastric myoelectric activity, organ distribution, pharmacokinetics, video capsule enteroscopy

## Abstract

Gastrointestinal side effects of donepezil, including dyspepsia, nausea, vomiting or diarrhea, occur in 20–30% of patients. The pathogenesis of these dysmotility associated disorders has not been fully clarified yet. Pharmacokinetic parameters of donepezil and its active metabolite 6-*O*-desmethyldonepezil were investigated in experimental pigs with and without small intestinal injury induced by dextran sodium sulfate (DSS). Morphological features of this injury were evaluated by a video capsule endoscopy. The effect of a single and repeated doses of donepezil on gastric myoelectric activity was assessed. Both DSS-induced small intestinal injury and prolonged small intestinal transit time caused higher plasma concentrations of donepezil in experimental pigs. This has an important implication for clinical practice in humans, with a need to reduce doses of the drug if an underlying gastrointestinal disease is present. Donepezil had an undesirable impact on porcine myoelectric activity. This effect was further aggravated by DSS-induced small intestinal injury. These findings can explain donepezil-associated dyspepsia in humans.

## 1. Introduction

**Figure 1:**
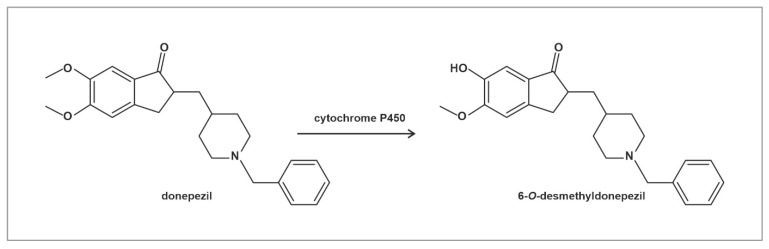
Structure of donepezil and its primary active metabolite 6-*O*-desmethyldonepezil. Donepezil is metabolized mainly by CYP 2D6 and 3A4.

Donepezil, a potent reversible cholinesterase inhibitor, is still being used as the preferred treatment for Alzheimer’s disease [1]. Donepezil is metabolized in the liver by cytochrome P450, producing various metabolites with similar half-lives to the unchanged parent drug [2]. From these metabolites, 6-*O*-desmethyldonepezil (Figure 1), as well as donepezil-N-oxide, also show activity towards cholinesterase [3,4,5].

Gastrointestinal side effects of donepezil, including dyspepsia, nausea, vomiting or diarrhea, occur in 20–30% of patients [6,7]. The pathogenesis of these dysmotility disorders has not been fully clarified yet.

In clinical practice, the majority of the recipients of donepezil treatment are the elderly. Aging has a significant impact on the gastrointestinal tract even in healthy seniors, including effects on the absorption of nutrients and drugs, motility, the mucosal defense system and immune function [8,9]. The function of the small bowel in the elderly can be further deteriorated by an underlying disease, like inflammatory disorders (e.g., celiac disease, inflammatory bowel disease, chronic gastrointestinal infections), atherosclerotic mesenteric ischemia, drug-induced small intestinal injury (e.g., non-steroidal anti-inflammatory drugs) and/or small intestinal bacterial overgrowth [10,11,12].

Experimental dextran sodium sulfate (DSS)-induced injury can affect the function and morphology of different parts of the gastrointestinal tract, including the stomach, small intestine and large bowel [13,14,15]. Experimental pigs can be used in preclinical studies due to their similar gastrointestinal functions compared with humans [16,17,18].

Our current study had three major aims. The first one was to evaluate pharmacokinetic parameters of donepezil and its major active metabolite 6-*O*-desmethyldonepezil in experimental pigs with or without small intestinal injury induced by DSS. The second aim was to evaluate morphological features of the DSS-induced injury by means of video capsule enteroscopy. The third aim was to assess the effect of a single dose and repeated doses of donepezil on gastric myoelectric activity in experimental pigs.

## 2. Results

### 2.1. Absorption and Distribution of Donepezil and 6-O-desmethyldonepezil

**Figure 2:**
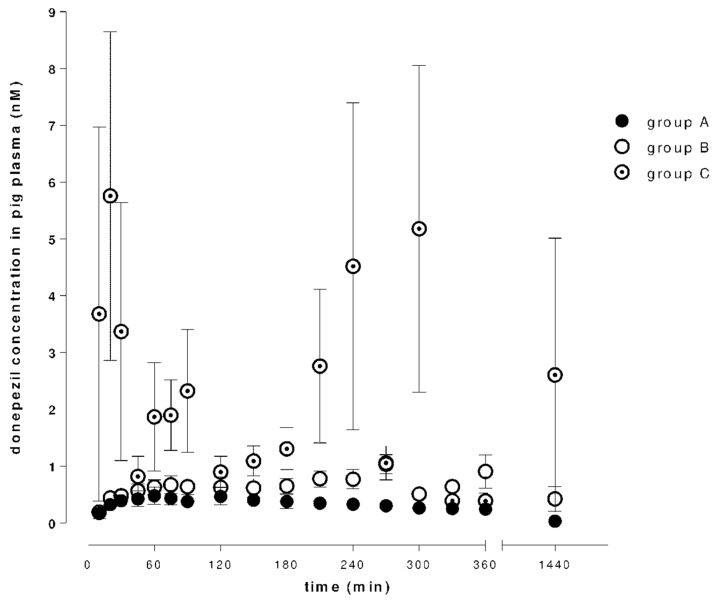
Concentrations of donepezil in porcine plasma after a single intragastric dose of donepezil (5 or 10 mg; day 1), and after a dextran sodium sulfate (DSS)-induced gastrointestinal injury (10 mg dose of donepezil; day 8).

**Figure 3:**
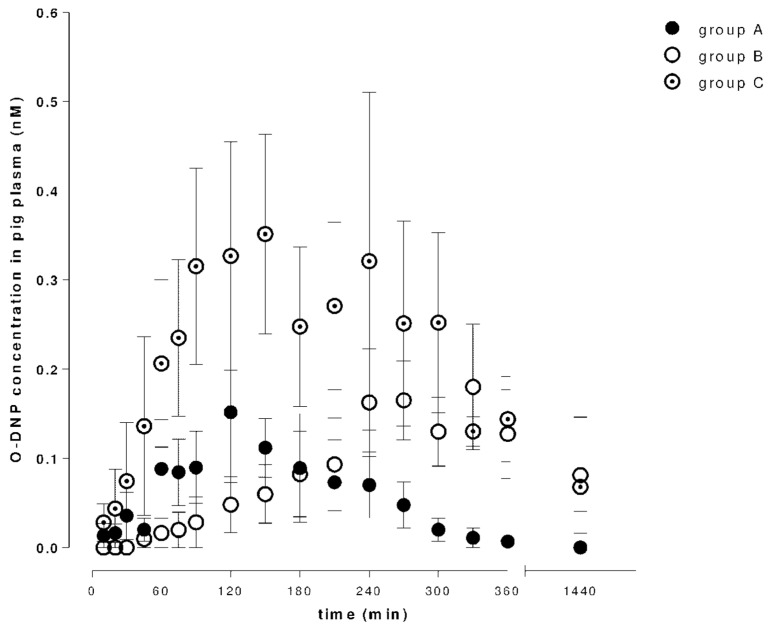
Concentrations of 6-*O*-desmethyldonepezil (O-DNP) in porcine plasma after a single intragastric dose of donepezil (5 or 10 mg; day 1), and after a dextran sodium sulfate (DSS)-induced gastrointestinal injury (10 mg dose of donepezil; day 8).

Figure 2 and Figure 3 show time–concentration curves of donepezil or 6-*O*-desmethyldonepezil in plasma after 5 mg (group A), 10 mg (group B) and 10 mg + DSS (group C) intragastric administrations.

There was a dose dependency in donepezil (C_max_ 0.55 ± 0.13 ng/mL for a 5 mg dose and 1.25 ± 0.19 ng/mL for a 10 mg dose), but administration of a double dose did not result in a two-fold increase in plasma 6-*O*-desmethyldonepezil concentrations. Pharmacokinetic analysis found a significant difference in donepezil plasma concentration in group C. Comparing AUC_last_ values of a 10 mg dose, the ratios were approximately twice as high in the case of DSS-induced intestinal injury in both tested compounds. T_max_ increased with a higher dose of donepezil. In group C, the T_max_ was increased in the case of donepezil and decreased in the case of 6-*O*-desmethyldonepezil. The pharmacokinetic results are summarized in Table 1.

Table 2 gives data on organ distribution. Animals received donepezil for 7 days (5 mg per day, in a dietary bolus) with an extra intragastric dose on day 8 (10 mg). The plasma, body fluids and organs were collected 24 h after the last donepezil dose. For comparison of bio-distributive relationships of donepezil and 6-*O*-desmethyldonepezil in particular tissues organ/plasma, ratios are provided.

### 2.2. Capsule Enteroscopy

**Figure 4:**
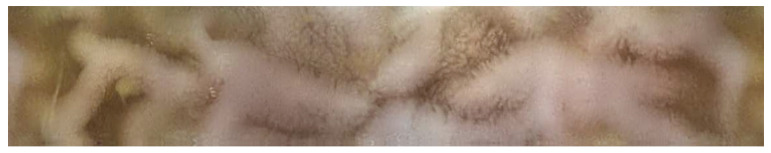
Video capsule enteroscopy. Normal porcine jejunum.

**Figure 5:**
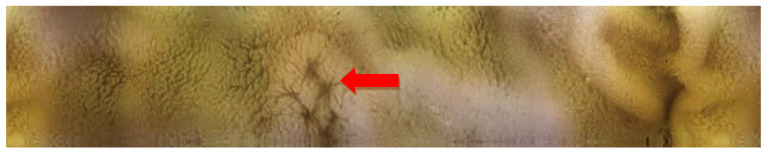
Video capsule enteroscopy. Peyer’s patches of the normal porcine ileum (arrow).

**Figure 6:**
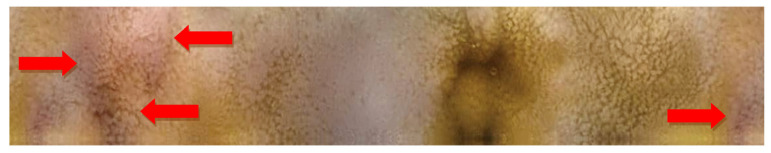
Video capsule enteroscopy. Porcine ileum. Focal mucosal erythema (arrows).

**Figure 7:**
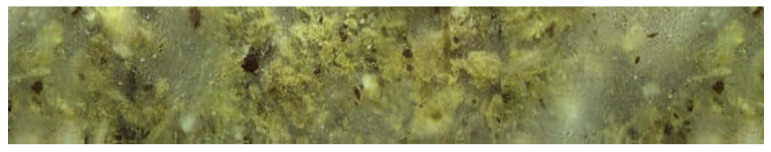
Video capsule enteroscopy. Porcine ileum with remnants of digested food.

One capsule endoscope was broken by an animal. The remaining five video recordings were available for evaluation. Transit time (from the pylorus to the ileo-cecal valve) ranged from 228 to 520 min (mean 347 ± 128; median 324 min). No severe pathology (e.g., ulcers) nor intestinal bleeding were found in any studied animal. Four animals revealed normal enteroscopy (Figure 4 and Figure 5), one pig had multifocal small areas of mucosal erythema in the ileum (Figure 6). There were remnants of digested food in the terminal ileum in one case (Figure 7) which made an appropriate evaluation of this area impossible.

Transit time did not correlate with peak plasma concentrations of donepezil and its active metabolite 6-*O*-desmethyldonepezil. Animals with a shorter transit time (228 and 229 min) had significantly lower average plasma concentrations of donepezil (mean: 1.68 ± 3.67; median 0.60, interquartile range 0.39–0.84 ng/mL) compared to animals with a longer transit time (433 and 520 min; mean: 10.41 ± 20.02; median 1.60, interquartile range 0.94–10.80 ng/mL), *p* < 0.001. Such a difference was not found in plasma concentrations of 6-*O*-desmethyldonepezil (*p* = 0.346; power of the performed test: 0.678).

### 2.3. Electrogastrography

**Figure 8:**
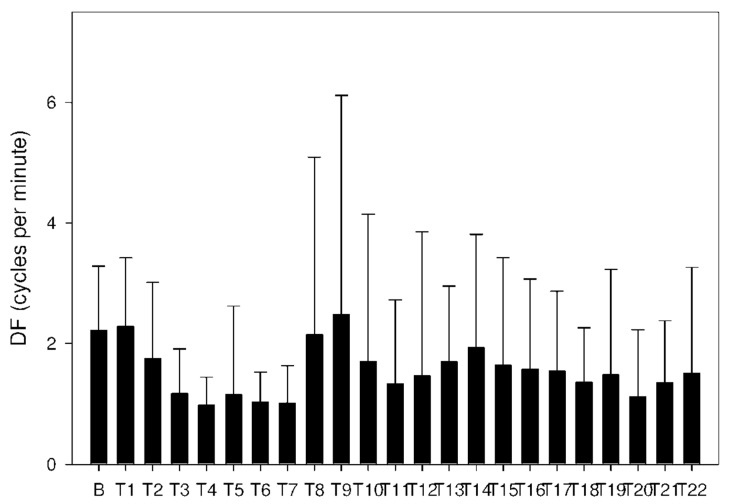
Electrogastrography. Dominant frequency after a single intragastric dose of donepezil (5 mg; group A).

**Figure 9:**
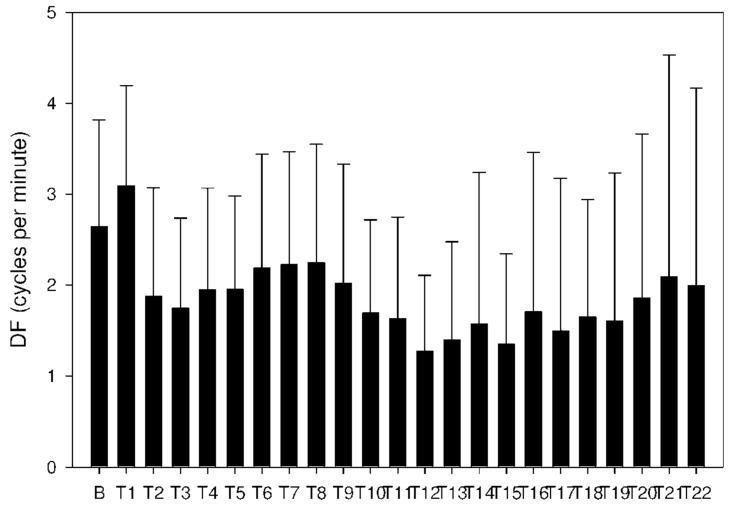
Electrogastrography. Dominant frequency after a single intragastric dose of donepezil (10 mg; group B).

Altogether, 6210 one-minute electrogastrography (EGG) recordings were evaluated, each in dominant frequency (DF), power and power ratio. A total of 38 outliers (0.6% of all recordings; from various time intervals of different animals) were excluded from the final evaluation of power and power ratio. Groups A and B revealed similar trends. DF decreased from basal values in groups A and B after 60 min, as shown in Table 3 and Figure 8 and Figure 9.

**Figure 10:**
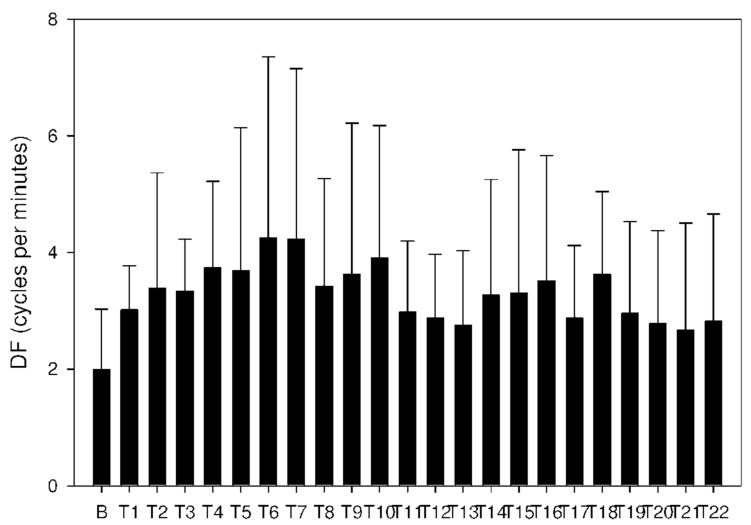
Electrogastrography. Dominant frequency after an intragastric administration of 10 mg donepezil (on day 8; group C) with preceding 7-day administration of donepezil (5 mg per day) and dextran sodium sulfate (10 g per day).

**Figure 11:**
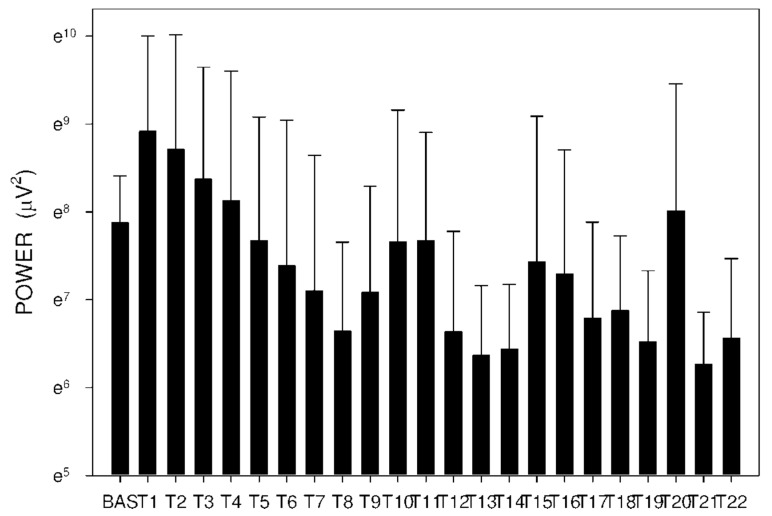
Electrogastrography power after a single intragastric dose of 5 mg of donepezil (group A). Outliers omitted. Y axis: natural log scale.

**Figure 12:**
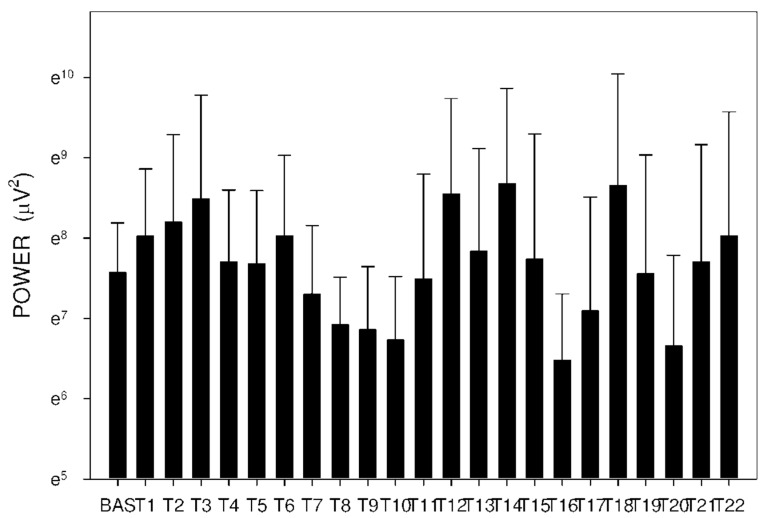
Electrogastrography power after a single intragastric dose of 10 mg of donepezil (group B). Outliers omitted. Y axis: natural log scale.

**Figure 13:**
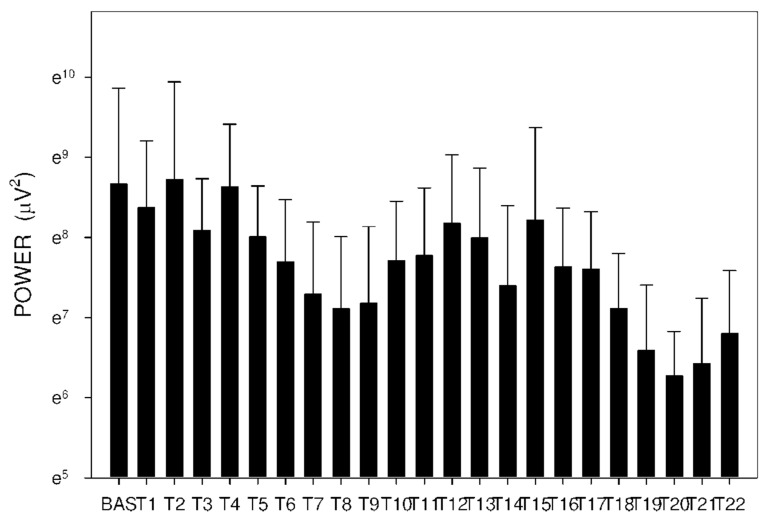
Electrogastrography power after an intragastric administration of 10 mg donepezil (on day 8; group C) with preceding 7-day administration of donepezil (5 mg per day) and dextran sodium sulfate (10 g per day). Outliers omitted. Y axis: natural log scale.

**Figure 14:**
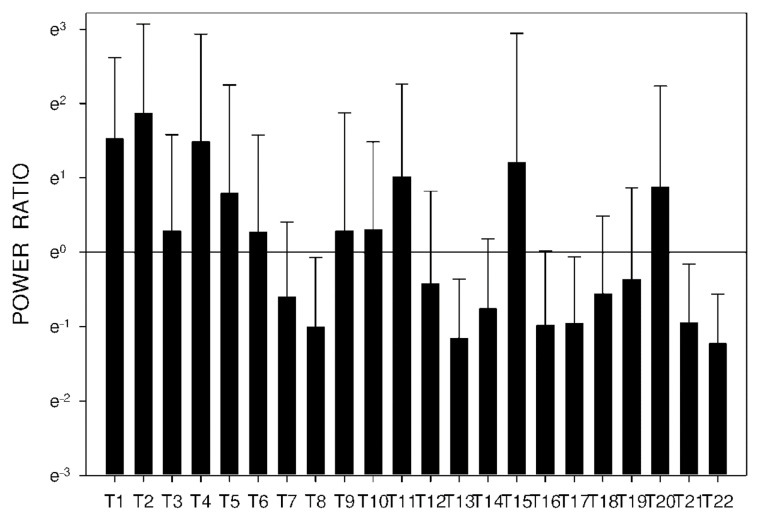
Electrogastrography power ratio after a single intragastric dose of 5 mg of donepezil (group A). Outliers omitted. Y axis: natural log scale.

**Figure 15:**
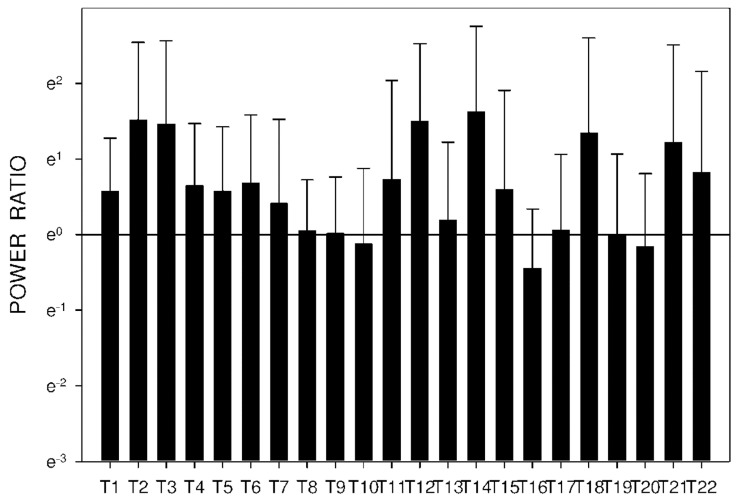
Electrogastrography power ratio after a single intragastric dose of 10 mg of donepezil (group B). Outliers omitted. Y axis: natural log scale.

**Figure 16:**
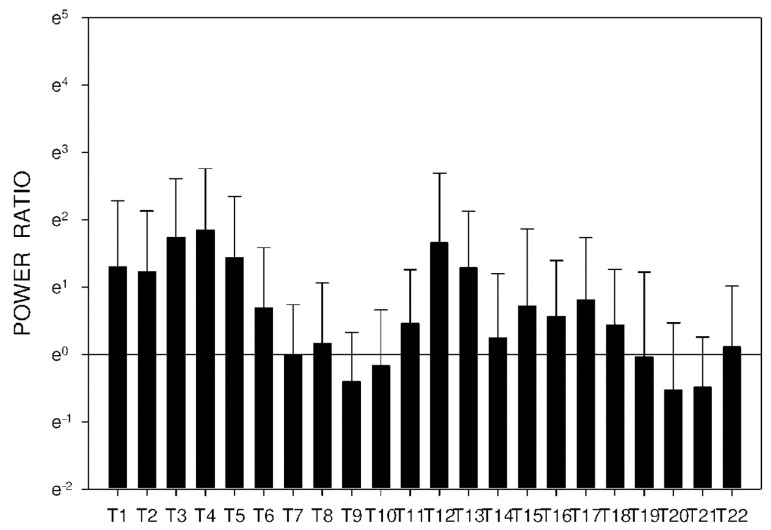
Electrogastrography power ratio after an intragastric administration of 10 mg donepezil (on day 8; group C) with preceding 7-day administration of donepezil (5 mg per day) and dextran sodium sulfate (10 g per day). Outliers omitted. Y axis: natural log scale.

In group C, DF increased from basal values significantly after 105 min. Higher DF was then sustained for the following 165 min, as shown in Table 3 and Figure 10. Power and power ratio had a corresponding pattern (Table 4, Figure 11, Figure 12, Figure 13, Figure 14, Figure 15 and Figure 16).

In group A, the power ratio decreased from its highest values thirty minutes after the drug administration to low levels after an interval of 120 min. Group B showed a similar profile, as shown in Figure 14 and Figure 15. In group C, the power ratio dropped significantly from higher values 30 min from the start to low levels after 120 min. The power ratio rose again after the following 60 min, terminating with decreased values after 315 min, as shown in Figure 16.

## 3. Discussion

Our current study brought new important insights into the pharmacokinetics of donepezil and its active metabolite 6-*O*-desmethyldonepezil in experimental pigs. To our best knowledge, this is the first porcine study that evaluates different factors influencing pharmacokinetics of donepezil and the first study evaluating its impact on the gastric myoelectric activity in experimental pigs.

The majority of donepezil is excreted through urine without any change [19], however, a portion of donepezil also undergoes several biotransformation steps while it is in the body. The metabolite 6-*O*-desmethyldonepezil has been considered to be as active as its parent molecule [2]. Still, another minor metabolite, donepezil-N-oxide, has also been proven to affect acetylcholinesterase [4]. We decided to assess 6-*O*-desmethyldonepezil levels, as the primary major active metabolite of donepezil, in the plasma and selected organs. Following administration of ^14^C-labeled donepezil in human healthy volunteers, plasma radioactivity, expressed as a percentage of the administered dose, was present primarily as intact donepezil (53%) and as 6-*O*-desmethyl donepezil (11%), which has been reported to inhibit acetylcholinesterase to the same extent as donepezil in vitro and was found in the plasma at concentrations equal to about 20% of donepezil [19].

Plasma concentrations after a single intragastric administration in our study were dose dependent for donepezil but not for 6-*O*-desmethyldonepezil. However, T_max_ was longer for both substances after the intragastric dose of donepezil had been doubled. In humans, the maximum plasma concentration is reached within 3–4 h after the oral donepezil administration, which is in line with our results of 1.5–4.5 h in our study. Of note, T_max_ was delayed for both substances with an increased dose. The biological half-time is 50–70 h, so the steady state is set within a few days in humans [5,20]. Underlying gastrointestinal disease and/or injury to the small bowel can have a significant impact on the pharmacokinetics and bioavailability of several drugs. According to our previous study [13], we decided to use lower doses of DSS, so that only functional, but not morphological, changes of the gastrointestinal tract were induced. Major DSS-induced structural pathology was excluded by capsule endoscopy. Porcine DSS-induced small intestinal injury led to significantly higher plasma concentrations of donepezil with a maximum at 5 h after administration of the last dose of donepezil. A breakthrough finding is the effect of transit time (time from the pylorus to ileo-cecal valve) on the plasma concentration of donepezil: a prolonged transit time of the video capsule was associated with significantly higher concentrations of donepezil. Donepezil has four major metabolites, two of them (6-*O*-desmethyldonepezil, donepezil-N-oxide) are pharmacologically active [5,20]. This fact can explain why those changes in plasma concentrations mentioned above were not found for 6-*O*-desmethyldonepezil.

Despite extensive studies regarding the improvement of the passage of donepezil across the blood–brain barrier [21,22,23], basic human distribution studies are still lacking. The first estimations were based on human acetylcholinesterase activity changes in cerebrospinal fluid (CSF). The activity between the doses fluctuated significantly [24]. Donepezil levels in CSF have been estimated as being ten times lower than in plasma [19,25,26]. The only clinical study with direct assessment of CSF donepezil levels with Alzheimer’s disease patients in a steady state showed that plasma concentrations in patients who take 10 mg donepezil per day orally correspond to approximately 40 ng/mL 12 h after application, and 30 ng/mL after 24 h; CSF concentrations were 5.2 ng/mL 12 h after application and 7.5 ng/mL after 24 h [7]. A significantly lower donepezil concentration was found in CSF when compared to plasma. On the other hand, donepezil accumulation in brain tissue was found in mice, rats and rabbits [27].

Clinical studies assessing the distribution of donepezil into the selected human brain segments are also not available. Although some preclinical and clinical data on donepezil have been published, details on human brain pharmacokinetics and pharmacodynamics are only estimated. We used the porcine model for its resemblance to humans. Donepezil accumulated in all brain regions, reaching at least twice the plasma concentrations; the concentrations resembled those in peripheral fat tissue. The pituitary is not protected by the blood–brain barrier. Higher donepezil concentration in the pituitary compared the other brain regions suggests a role of the blood–brain barrier protection and active efflux mechanisms. In contrast to memantine, where the highest concentrations were found in the hippocampus and medulla [15], donepezil accumulated mainly in the medulla and medulla oblongata.

6-*O*-desmethyldonepezil accounts for approximately 26–27% of donepezil in human plasma [28,29]. In the current study on pigs, 6-*O*-desmethyldonepezil reached 24–25% of donepezil concentrations; we may thus assume that the pig metabolic kinetics are similar to humans and so the ratios in peripheral tissues and brain segments may be as well. Brain concentrations differed depending on the region. These are the results conveyed as the percentage of plasmatic concentrations (particular regions compared to plasma): medulla 105%, medulla oblongata 100%, front lobe 75%, cerebellum 76%, hippocampus 83% and diencephalon 83%. These results indicate that 6-*O*-desmethyldonepezil plays a significant role in the pharmacodynamic effect of donepezil in the brain. The lowest ratio (64%) was in the pituitary gland. This may be due to its direct connection with the blood. These data also suggest that there might be an intense metabolism of donepezil in the brain tissue.

In the gastrointestinal tract, the highest concentration was found in the proximal jejunum, the place of donepezil absorption [29]. The concentration of donepezil in the intestinal wall decreased with increasing distance from the stomach. A markedly higher concentration of donepezil was found in the liver, which indicates a possible first pass effect, the proportion of active metabolite accounted for was 34%. In many aspects, the pharmacokinetic profile of donepezil follows similar patterns to memantine after intragastric administration [15], where highly perfused organs and organs of elimination (lung, kidney, liver) contained high concentrations of the drug. Donepezil also accumulated in the spleen. The binding of donepezil to acetylcholinesterase on erythrocyte surfaces might explain this phenomenon, since the spleen acts as a final destination of erythrocytes. A low concentration was found in the heart, skin, muscle and fat; the accumulation of donepezil was only four times higher compared to plasma. In some peripheral tissues, the concentration of 6-*O*-desmethyldonepezil was higher compared to donepezil (fat 114%, skin 151% and muscle 158%). The most likely explanation is the active taking up of 6-*O*-desmethyldonepezil from the blood stream by these tissues.

Donezepil caused an unfavorable effect on porcine myoelectric activity, assessed by gastric power. This can indeed be a plausible explanation for donepezil-associated dyspepsia in humans. DSS caused an undesirable increase in dominant frequency and decreased EGG power. This can be a proof that an underlying disease in elderly humans may aggravate the effect of donepezil on gastric myoelectric activity.

We are aware of possible limitations of this study. Higher plasma concentrations of donepezil in group C (with a DSS-induced small intestinal injury), which were obtained on day 8, could have been influenced, at least partially, by additional daily doses of donepezil (5 mg per day, for 7 days).

## 4. Methods

### 4.1. Animals

Eighteen experimental adult female pigs (*Sus scrofa* f. *domestica*, hybrids of Czech White and landrace breeds; 3 months old; mean weight 38.7 ± 1.0, median 39.0 kg) were enrolled in the study. The animals were purchased from a certified breeder (Stepanek, Dolni Redice, Czech Republic; SHR MUHO 2050/2008/41). The pigs were housed in an accredited vivarium (temperature 21 ± 1 °C, 12-h light/dark cycle; Faculty of Military Health Sciences, Hradec Kralove, Czech Republic). All animals were fed with a standard assorted A1 food (Ryhos, Novy Rychnov, Czech Republic) with equal amounts twice a day and had free access to drinking water.

### 4.2. Design of the Study

Experimental pigs were randomly divided into three group (six animals per group). All procedures were carried out under general anesthesia. Intramuscular injections of ketamine (20 mg per kg; Narkamon, Spofa, Prague, Czech Republic) and azaperone (2.2 mg per kg; Stresnil, Janssen Animal Health, Saunderton, UK) were used as an introduction to anesthesia in all animals. Intravenous infusion of propofol (AstraZeneca AB, Stockholm, Sweden) was used for subsequent maintenance of general anesthesia. Two vital signs were monitored, heart rate and pulse oximetry, and were used to secure the experiments and to assess possible myocardial effects of donepezil.

Pharmacokinetics of donepezil hydrochloride and 6-*O*-desmethyldonepezil were investigated after a single intragastric dose of donepezil (5 mg in group A and 10 mg in group B; all in a 50 mL water suspension). The whole dose of donepezil was administrated into the stomach endoscopically, using a GIF-Q180 video-gastroscope (Olympus Optical Co, Tokyo, Japan) dedicated for animal use only. The third group (C) received donepezil for 7 days (5 mg per day; in a dietary bolus) with an extra intragastric dose on day 8 (10 mg). DSS was administered to overnight fasting animals in another dietary bolus in the morning for 7 days (10 g per day). Donepezil hydrochloride was purchased from EGIS Pharmaceuticals PLC (Budapest, Hungary). Dextran sodium sulfate salt was purchased from Sigma-Aldrich (Prague, Czech Republic).

Electrogastrography was recorded in all three groups of animals (see Section 4.3). Wireless capsule endoscopy was performed in group C on day 8 (see Section 4.4).

Concentrations of donepezil and its active metabolite 6-*O*-desmethyldonepezil were measured in porcine plasma which was collected after a single intragastric dose (groups A and B) and after repeated administration (on day 8; group C). Control blank and additional blood samples were drawn into heparinized tubes from a venous catheter at precisely defined time intervals up to 24 h following the donepezil intragastric administration. The blood samples (1 mL whole blood/time interval) were obtained at defined time intervals: 0, 10, 20, 30, 45, 60, 75, 90, 120, 150, 180, 210, 240, 270, 300, 330, 360 min and 24 h. Plasma was prepared by centrifugation (1050× *g*, 10 min, 25 °C, Centrifuge Z 206 A, Hermle, Germany) which followed the sampling immediately and samples were frozen at −80 °C prior to the analysis.

For the study of organ distribution in group C, the pigs were exsanguinated in the morning on day 9 and immediate autopsy was performed. The blood and selected organs (heart, lung, muscle, fat, spleen, liver, kidney, gastric and intestinal wall, selected parts of the brain) were extracted and homogenized. For the elimination study, urine directly from the bladder and bile from the gallbladder were removed. Concentrations of donepezil and its primary active metabolite 6-*O*-donepezil were determined via high-performance liquid chromatography coupled with mass spectrometry in both plasma and organ homogenates.

### 4.3. Electrogastrography

Our original method of porcine surface electrogastrography (EGG) has already been published elsewhere [15,30,31]. Briefly, six active self-adhesive electrodes were placed on the upper part of the abdomen, the 7th electrode (basal) was placed to the left of the middle of the sternum. A special abdominal belt (respiratory sensor) was used to identify possible artefacts caused by breathing and body movements. Gastric myoelectric activity was investigated in fasting animals in the morning on day 1 (groups A and B) and day 8 (group C) using an EGG stand (MMS, Enschede, the Netherlands). All EGGs were performed with propofol general anesthesia. Basal (15 min) and study recordings (330 min) were evaluated by MMS software (version 8.19). Running spectral analysis was used for a standard evaluation of EGG. Results were expressed as dominant frequency of gastric slow waves (DF; cycles per minute, cpm), power analysis (areas of amplitudes; μV^2) and power ratio assessment: fraction of the areas of amplitudes after (numerator) and before (denominator) the study drugs.

### 4.4. Capsule Endoscopy

Our group has established our own method of wireless capsule endoscopy in experimental pigs [32,33]. In this study, we used a CapsoCam system (CapsoVision, Saratoga, NY, USA) that combines four camera images into a seamless 360° panoramic view of the small bowel. Capsules were introduced endoscopically into the duodenum by means of a special delivery system (AdvanCE, US Endoscopy, Mentor, CA, USA). After the examination had been completed, endoscopic capsules were captured and image data were downloaded and evaluated by dedicated software.

### 4.5. LC–MS Analysis of Donepezil and 6-O-desmethyldonepezil

The system used in this study was Dionex Ultimate 3000 UHPLC: RS LPG quaternary pump, RS column compartment, RS autosampler, diode array detector, Chromeleon (version 7.2.9 build 11323) software (Thermo Fisher Scientific, Germering, Germany) with a Q Exactive Plus Orbitrap mass spectrometer with Thermo Xcalibur (version 3.1.66.10.) software (Thermo Fisher Scientific, Bremen, Germany). Detection was carried out by mass spectrometry in a positive mode. Settings of the heated electrospray source were: spray voltage 3.5 kV, capillary temperature: 300 °C, sheath gas: 55 arbitrary units, auxiliary gas: 15 arbitrary units, spare gas: 3 arbitrary units, probe heater temperature: 250 °C, max spray current: 100 µA, S-lens RF level: 50.

#### 4.5.1. Chemicals and Reagents

Solvents (methanol, acetonitrile, phosphate-buffered saline, acetone and other common chemicals) were purchased from Sigma-Aldrich (Hamburg, Germany). Solvents for extraction and the chromatographic part of the experiment were supplied in liquid chromatography with tandem mass spectrometry (LC–MS) grade.

#### 4.5.2. Sample Preparation

Organs were weighed, four times their weight of PBS was added and, subsequently, they were homogenized by a T-25 Ultra Turrax disperser (IKA, Staufen, Germany), transferred to 5 mL polypropylene microcentrifuge tubes, sonicated by a UP 50H needle homogenizer (Hielscher, Teltow, Germany) and stored at −80 °C prior to the extraction.

A total amount of 290 µL of plasma was spiked with 10 µL of internal standard (IS; D7-donepezil in methanol), so that the final concentration was 5nM. This was vortexed quickly and 1000 µL of ethyl acetate were added. The samples were then shaken for 10 min (1200 RPM, Wizard Advanced IR Vortex Mixer, Velp Scientifica, Usmate, Italy) and centrifuged (12,000 RPM, 5 min, Universal 320 R centrifuge, Hettich, Tuttlingen, Germany). In total, 700 µL of supernatant were transferred to a 2 mL polypropylene micro-tube. Afterwards, 1000 µL of ethyl acetate were added and extraction was repeated, and supernatants were combined and evaporated in a CentriVap concentrator to dryness (Labconco Corporation, Kansas City, MO, USA). Prior to the analysis, samples were reconstituted in 50 µL of 50% (*v*/*v*) acetonitrile. Calibration samples were prepared by spiking 270 µL of blank matrix with 10 µL of donepezil (Tokyo Chemical Industry, Tokyo, Japan), 10 µL of 6-*O*-desmethyldonepezil (Syncom, Groningen, the Netherlands) methanolic solution (final concentrations ranging from 0.01 to 50 nM) and 10 µL of internal standard (IS) (D7-donepezil; Toronto Research Chemicals, North York, ON, Canada; in methanol; final concentration 5 nM). Subsequently, this was vortexed and extracted as above.

Finally, 190 µL of organ homogenate were spiked with 10 µL of internal standard, vortexed and 1000 µL of acetone were added. The samples were then shaken for 20 min (1200 RPM) and centrifuged (10,840× *g*, 5 min). In total, 800 µL of supernatant were transferred to a 2 mL polypropylene microtube and evaporated to dryness. Prior to the analysis, samples were reconstituted in 100 µL of 50% (*v*/*v*) ACN. Calibration samples were prepared by spiking 170 µL of organ homogenate with 10 µL of donepezil, 10 µL of 6-*O*-desmethyldonepezil methanol solution (final concentrations ranging from 0.1 to 1000 nM according to organ) and 10 µL of IS (final concentration 5 nM) and then were vortexed and extracted as above.

#### 4.5.3. LC–MS/MS Analysis

Chromatography was performed by a modified method described by Zdarova Karasova et al. [34]. Data were obtained by a reverse phase gradient elution method with a C18 column (Luna Omega polar C18, 2.1 × 50 mm, 1.6 µm, Phenomenex, Japan) with mobile phase A: 0.1% (*v*/*v*) formic acid in ultrapure water of ASTM type I (resistance 18.2 MΩ.cm at 25 °C) prepared by Barnstead Smart2Pure 3 UV/UF apparatus (Thermo Fisher Scientific, Bremen, Germany) and mobile phase B: 0.1% (*v*/*v*) formic acid in LC–MS grade acetonitrile and methanol 50/50 (*v*/*v*). Mobile phase flow was set to 0.6 mL × min^−1^ and the gradient method was as follows: started with 5% B isocratic flow for 0.5 min, then gradient of B for 2.5 min from 5 to 100% B, then the flow was isocratic for 1 min of 100% B and then the system went back to 5% B and equilibrated for 2 min. Total run time of the method was 6 min. The column was heated to 40 °C. The injection volume was 5 µL. Samples were analyzed by a mass spectrometer in a parallel reaction monitoring setting. Settings for donepezil, 6-*O*-desmethyldonepezil and D7-donepezil are in Table 5.

The calibration curve for plasma had 12 points within a range 0.01 to 50 nM. Calibration curves for organ homogenates had 7 points: skin (0.5–500 nM), adipose tissue (0.5–500 nM), bile (1–1000 nM), urine (1–1000 nM); 8 points: spinal cord, medulla oblongata, cerebellum, diencephalon, pituitary gland, frontal lobe, hippocampus (0.1–500 nM), muscle, heart (0.1–500 nM); 9 points: liver, lungs, spleen, kidneys (0.1–1000 nM) and intestine (0.1–1000 nM).

### 4.6. Statistical Analysis

Data were statistically treated by means of descriptive statistics, a non-paired *t*-test, a Mann–Whitney rank sum test and Pearson product moment correlation using SigmaStat software (Version 3.1, Jandel Corp, Erkrath, Germany).

The time-dependent changes of donepezil and 6-*O*-desmethyldonepezil in each sample were recalculated to real concentrations (µg/mL in body fluids and µg/g in tissues) using the calibration curve in GraphPad Prism version 6.05 (GraphPad Software, San Diego, CA, USA). The pharmacokinetic profile is calculated as mean ± SEM. For a standard non-compartmental analysis, Kinetica software, version 4.0 (InnaPhase Corporation, Thermo Fisher Scientific, Waltham, MA, USA) was used. Maximum concentration (C_max_) and the time to the maximum concentration (T_max_) were determined directly from the measured data. The area under the mean plasma concentration–time curve from time zero to the last measured concentration (AUC_last_) was determined.

### 4.7. Ethics

The project was approved by the Institutional Review Board of the Animal Care Committee of the University of Defence, Faculty of Military Health Sciences, Hradec Kralove, Czech Republic (Protocol No. MO 171673/2019-684800). The study was conducted in accordance with the policy for experimental and clinical studies [35]. Animals were held and treated in conformity with the European Convention for the Protection of Vertebrate Animals [36] and in accordance with the ARRIVE Guidelines [37].

## 5. Conclusions

In conclusion, both DSS-induced small intestinal injury and prolonged small intestinal transit time caused higher plasma concentrations of donepezil in experimental pigs. This has an important implication for clinical practice in humans, with a need to reduce doses of the drug in the case of an underlying gastrointestinal disease. Donepezil had an undesirable impact on porcine myoelectric activity. This effect was further aggravated by DSS-induced small intestinal injury. These findings can explain donepezil-associated dyspepsia in humans.

## Figures and Tables

**Table 1:** Pharmacokinetic parameters. Pharmacokinetic parameters after a single intragastric dose of donepezil (5 or 10 mg; groups A and B), and after a DSS-induced gastrointestinal injury (10 mg of donepezil; group C). All values are expressed as mean ± SEM. Cmax is defined as maximum donepezil or 6-*O*-desmethyldonepezil plasma concentrations; Tmax is time of Cmax; AUClast means the area under the concentration–time curve from zero time up to the last measured concentration.

Groups	Donepezil			6-*O*-desmethyldonepezil		
	Cmax ng/mL	Tmax min	AUClast min.ng/mL	Cmax ng/mL	Tmax min	AUClast min.ng/mL
A	0.55 ± 0.13	87.50 ± 14.32	232.5 ± 41.72	0.21 ± 0.007	125.0 ± 13.07	22.1 ± 8.50
B	1.25 ± 0.19	267.5 ± 38.79	877.67 ± 234.8	0.21 ± 0.06	275.0 ± 24.91	121.52 ± 69.58
C	12.08 ± 2.28	306.67 ± 209.24	1913.67 ± 872.77	0.52 ± 0.15	157.5 ± 35.66	208.3 ± 83.1

DSS: dextran sodium sulfate; SEM: standard error of the mean.

**Table 2:** Concentrations of donepezil and 6-O-desmethyldonepezil. Concentrations of donepezil and 6-*O*-desmethyldonepezil (*O*-DNP) in porcine plasma and selected organs/body fluids collected 24 h after the last intragastric administration of 10 mg donepezil (on day 8) with preceding 7-day administration of donepezil (5 mg per day) and dextran sodium sulfate (10 g per day).

Tissue	Donepezil (nM)	Ratio	*O*-DNP (nM)	Ratio
	Mean ± SEM		Mean ± SEM	
plasma	1.77 ± 0.35	1.00	0.43 ± 0.10	1.00
stomach	72.16 ± 15.89	40.76	1.45 ± 0.22	3.37
proximal jejunum	370.53 ± 112.94	209.34	17.25 ± 4.02	40.12
mid jejunum	104.61 ± 68.27	59.10	4.33 ± 0.88	10.07
ileum	14.42 ± 1.30	8.15	3.96 ± 1.19	9.21
colon	14.61 ± 1.54	8.25	6.60 ± 4.69	15.35
muscle	6.26 ± 0.29	3.54	9.91 ± 0.50	23.05
fat	5.68 ± 0.74	3.20	6.46 ± 2.25	15.02
skin	5.22 ± 0.68	2.95	7.88 ± 0.94	18.33
spleen	15.34 ± 1.74	8.66	3.26 ± 0.27	7.58
liver	127.14 ± 8.57	71.83	44.33 ± 5.31	103.09
kidney	25.56 ± 3.03	14.44	21.67 ± 3.44	50.40
heart	4.38 ± 0.21	2.48	1.00 ± 0.17	2.33
lung	20.73 ± 1.13	11.71	3.73 ± 0.43	8.67
medulla	7.99 ± 1.22	4.51	8.36 ± 1.68	19.44
medulla oblongata	7.02 ± 0.64	3.97	7.03 ± 0.97	16.35
front lobe	5.21 ± 0.32	2.94	3.95 ± 0.25	9.19
cerebellum	4.73 ± 0.20	2.67	3.60 ± 0.15	8.37
pituitary gland	13.12 ± 0.99	7.41	8.40 ± 0.48	19.54
hippocampus	5.22 ± 0.44	2.95	4.31 ± 0.56	10.02
diencephalon	4.58 ± 0.37	2.58	3.82 ± 0.20	8.88
urine	45.88 ± 9.68	25.92	62.90 ± 12.48	146.28
bile	601.63 ± 112.12	339.90	444.35 ± 132.62	1033.37

*O*-DNP: 6-*O*-desmethyldonepezil; SEM: standard error of the mean.

**Table 3:** Electrogastrography. Dominant frequency (cycles per minute).

Period	A: Donepezil 5 mg Mean ± Std Dev Median IQR	B: Donepezil 10 mg Mean ± Std Dev Median IQR	C: Donepezil + DSS Mean ± Std Dev Median IQR	Significance *p* Value
Basal	2.2 ± 1.1	2.6 ± 1.2	2.0 ± 1.0	A-B: NS
	3.1	3.1	2.2	A-C: NS
	1.1–3.1	1.2–3.5	0.9–3.1	B-C: *p* < 0.001
T1	2.3 ± 1.1	3.1 ± 1.1	3.0 ± 0.8	A-B: *p* < 0.001
	2.8	3.3	3.3	A-C: *p* < 0.001
	0.9–3.3	2.6–3.5	3.1–3.5	B-C: NS
T4	1.0 ± 0.5	1.9 ± 1.1	3.7 ± 1.5	A-B: *p* < 0.001
	0.9	1.4	3.3	A-C: *p* < 0.001
	0.7–1.2	0.9–2.8	3.3–4.0	B-C: *p* < 0.001
T7	1.0 ± 0.6	2.2 ± 1.2	4.2 ± 2.9	A-B: *p* < 0.001
	0.9	2.6	3.1	A-C: *p* < 0.001
	0.7–1.2	1.2–3.1	2.8–7.0	B-C: *p* < 0.001
T9	2.5 ± 3.6	2.0 ± 1.3	3.6 ± 2.6	A-B: *p* = 0.011
	0.9	1.4	3.1	A-C: *p* < 0.001
	0.7–1.4	0.9–3.1	1.4–3.5	B-C: *p* < 0.001
T11	1.3 ± 1.4	1.6 ± 1.1	3.0 ± 1.2	A-B: *p* = 0.017
	0.9	1.3	3.3	A-C: *p* < 0.001
	0.7–1.4	0.7–2.6	2.6–3.5	B-C: *p* < 0.001
T15	1.6 ± 1.8	1.3 ± 1.0	3.3 ± 2.5	A-B: NS
	0.9	0.9	3.1	A-C: *p* < 0.001
	0.7–1.4	0.7–1.4	1.6–3.8	B-C: *p* < 0.001
T18	1.4 ± 0.9	1.6 ± 1.3	3.6 ± 1.4	A-B: NS
	1.2	1.2	3.3	A-C: *p* < 0.001
	0.9–1.4	0.7–2.1	3.3–4.0	B-C: *p* < 0.001
T22	1.5 ± 1.8	2.0 ± 2.2	2.8 ± 1.8	A-B: NS
	1.2	1.2	3.1	A-C: *p* < 0.001
	0.7–1.4	0.9–2.6	1.2–3.8	B-C: *p* < 0.001

DSS: dextran sodium sulfate; NS: not significant; IQR: interquartile range; Std Dev: standard deviation.

**Table 4:** Electrogastrography. Power (μV^2^); outliers omitted.

Period	A: Donepezil 5 mg Mean ± Std Dev Median IQR	B: Donepezil 10 mg Mean ± Std Dev Median IQR	C: Donepezil + DSS Mean ± Std Dev Median IQR	Significance *p* Value
Basal	2632 ± 1855	1938 ± 1658	5812 ± 13433	A-B: NS
	2153	1441	1438	A-C: NS
	1226–3616	825–2550	668–4139	B-C: NS
T1	7459 ± 14605	3068 ± 3963	4330 ± 5648	A-B: *p* = 0.001
	2772	1957	2262	A-C: NS
	1555–5703	592–3426	1392–5031	B-C: NS
T3	4337 ± 11106	4891 ± 12753	3254 ± 2955	A-B: NS
	570	579	2205	A-C: *p* < 0.001
	296–1748	200–1555	1370–3932	B-C: *p* < 0.001
T8	769 ± 1347	1015 ± 820	1227 ± 1780	A-B: *p* < 0.001
	408	803	745	A-C: *p* < 0.001
	205–712	414–1414	461–1064	B-C: NS
T10	2123 ± 7376	838 ± 1007	2234 ± 2457	A-B: NS
	342	587	739	A-C: *p* < 0.001
	209–817	168–1180	304–4497	B-C: *p* < 0.001
T12	762 ± 1623	5176 ± 11805	3569 ± 4773	A-B: *p* = 0.002
	346	703	1114	A-C: *p* < 0.001
	191–637	193–3181	378–5583	B-C: NS
T16	1475 ± 4554	652 ± 834	2068 ± 2235	A-B: NS
	319	269	1201	A-C: *p* < 0.001
	184–603	113–946	473–2740	B-C: *p* < 0.001
T20	3023 ± 9753	779 ± 1630	531 ± 393	A-B: *p* < 0.001
	423	215	425	A-C: NS
	208–1129	77–987	184–925	B-C: *p* = 0.007
T22	527 ± 426	3061 ± 11286	900 ± 1074	A-B: NS
	422	253	559	A-C: NS
	202–681	124–993	239–1264	B-C: NS

DSS: dextran sodium sulfate; IQR: interquartile range; NS: not significant; Std Dev: standard deviation.

**Table 5:** LC–MS/MS setting overview.

Compound	Parent Ion	Normalized Collision Energy	Selected Product Ion	t_R_ (min)
Donepezil	380.22	35	362.21	3.08
6-*O*-desmethyldonepezil	366.21	35	348.20	2.95
D7-donepezil	387.26	35	369.25	3.08

LC–MS/MS: liquid chromatography with tandem mass spectrometry; t_R_: retention time.

## Data Availability

All data generated or analyzed during this study are included in this article.

## Sample Availability

Samples of the compounds are not available from the authors.
